# Reliabilities of Genomic Prediction for Young Stock Survival Traits Using 54K SNP Chip Augmented With Additional Single-Nucleotide Polymorphisms Selected From Imputed Whole-Genome Sequencing Data

**DOI:** 10.3389/fgene.2021.667300

**Published:** 2021-07-19

**Authors:** Grum Gebreyesus, Mogens Sandø Lund, Goutam Sahana, Guosheng Su

**Affiliations:** Center for Quantitative Genetics and Genomics, Aarhus University, Tjele, Denmark

**Keywords:** young stock survival, genomic prediction, GWAS, whole-genome sequencing, recessive lethal alleles

## Abstract

This study investigated effects of integrating single-nucleotide polymorphisms (SNPs) selected based on previous genome-wide association studies (GWASs), from imputed whole-genome sequencing (WGS) data, in the conventional 54K chip on genomic prediction reliability of young stock survival (YSS) traits in dairy cattle. The WGS SNPs included two groups of SNP sets that were selected based on GWAS in the Danish Holstein for YSS index (YSS_SNPs, *n* = 98) and SNPs chosen as peaks of quantitative trait loci for the traits of Nordic total merit index in Denmark–Finland–Sweden dairy cattle populations (DFS_SNPs, *n* = 1,541). Additionally, the study also investigated the possibility of improving genomic prediction reliability for survival traits by modeling the SNPs within recessive lethal haplotypes (LET_SNP, n = 130) detected from the 54K chip in the Nordic Holstein. De-regressed proofs (DRPs) were obtained from 6,558 Danish Holstein bulls genotyped with either 54K chip or customized LD chip that includes SNPs in the standard LD chip and some of the selected WGS SNPs. The chip data were subsequently imputed to 54K SNP together with the selected WGS SNPs. Genomic best linear unbiased prediction (GBLUP) models were implemented to predict breeding values through either pooling the 54K and selected WGS SNPs together as one genetic component (a one-component model) or considering 54K SNPs and selected WGS SNPs as two separate genetic components (a two-component model). Across all the traits, inclusion of each of the selected WGS SNP sets led to negligible improvements in prediction accuracies (0.17 percentage points on average) compared to prediction using only 54K. Similarly, marginal improvement in prediction reliability was obtained when all the selected WGS SNPs were included (0.22 percentage points). No further improvement in prediction reliability was observed when considering random regression on genotype code of recessive lethal alleles in the model including both groups of the WGS SNPs. Additionally, there was no difference in prediction reliability from integrating the selected WGS SNP sets through the two-component model compared to the one-component GBLUP.

## Introduction

Young stock mortality represents a major economic loss for dairy farmers due, for instance, to fewer heifers available for replacement in the production system, fewer male calves for slaughter, higher veterinarian cost, and cost related to disposal of dead calf. In the Nordic countries, annual total loss due to dairy calf mortality (including stillbirth) is estimated to be approximately €70 million (Østerårs et al., 2007). In addition, young stock mortality poses a large animal welfare issue and threatens the public perceptions of the dairy industry.

Part of the variation in young stock mortality is genetic with reported heritability estimates ranging from 0.00 to 0.08 (e.g., Hansen et al., 2003; Fuerst-Waltl and Sørensen, 2010; Henderson et al., 2011). In the Nordic countries, young stock survival (YSS) in calves is included in the Nordic total merit (NTM) index (NAV).^1^ A challenge in the genetic evaluation for YSS traits is the low heritability leading to low prediction accuracies. Theoretically, there are possibilities to improve the reliability of genomic prediction models by incorporating causative variants (if known) or markers highly correlated with them (de Los Campos et al., 2013).

Genome-wide association studies (GWASs) based on sequence data have shown high power to identify putative causative variants and strong signals of association for various economic traits in cattle (Daetwyler et al., 2014; Sahana et al., 2014; Wu et al., 2017). Studies have shown that genomic prediction models incorporating single-nucleotide polymorphisms (SNPs) selected from whole-genome sequencing (WGS) data based on such GWASs lead to improved accuracy of prediction of breeding values for some traits. Brøndum et al. (2015) added quantitative trait loci (QTLs) from GWAS to genomic prediction models and achieved up to 5 percentage point increase in accuracy for milk production traits. Similarly, Liu et al. (2019) reported gains in prediction reliability for milk production traits in the Danish Jersey by integrating selected WGS variants with the 54K SNP chip. A GWAS by Wu et al. (2017) using WGS data reported interesting genomic regions across the *Bos taurus* autosome (BTA) significantly associated with the YSS index trait in the NTM index. Incorporating such WGS variants from GWASs might enable improvement of genomic prediction reliability for YSS traits. Additionally, the genetic underpinnings of young stock and calf mortality can be partly polygenic and partly due to deleterious effects of recessive lethal alleles (Gebreyesus et al., 2020). Several studies have reported haplotypes with harmful recessive effects on fertility and responsible for early embryonic lethality and stillbirth in various cattle breeds (e.g., VanRaden et al., 2011; Sahana et al., 2016; Hoff et al., 2017; Wu et al., 2019), which might have an important predictive ability for breeding values for YSS traits.

We hypothesize in this study that incorporation of WGS variants selected based on previous GWASs and variants within previously reported deleterious haplotypes might improve the reliability of genomic prediction for YSS traits. The objective of this study was therefore to investigate effects of integrating SNPs selected, based on previous studies, from imputed WGS data in the conventional 54K chip on genomic prediction of YSS traits in the Nordic Holstein cattle. Additionally, we also assessed the possibility of improving genomic prediction reliability for survival traits by considering in the prediction model the effect of SNPs located within recessive lethal haplotypes previously reported in the Nordic Holstein.

## Materials and Methods

### Ethics Approval Statement

All procedures to collect the DNA samples followed the protocols approved by the National Guidelines for Animal Experimentation and the Danish Animal Experimental Ethics Committee, and hence, no specific permission was required.

### Animals and Genotypes

A total of 6,558 Nordic Holstein bulls were genotyped with the Illumina Bovine SNP50 chip (54K, Illumina, Inc.). A reference population of 129,000 Holstein cows and bulls was also available for the imputation that were genotyped mostly with the EuroGenomics customized chip (Boichard et al., 2018) that included SNPs in the standard Illumina Bovine LD chip together with SNPs identified as causal mutation, functional annotation, or association with economic traits. The EuroGenomics customized chip that started with the standard LD chip (Boichard et al., 2018) is updated every year with selected variants and currently includes 70K SNPs including most of the variants in the conventional 54K chip along with additional selected SNPs. A total of 1,754 selected WGS SNPs, selected by GWAS in Denmark–Finland–Sweden dairy cattle populations (DFS_SNPs), are included in the EuroGenomics chip. The DFS_SNPs were peaks of QTL detected from imputed WGS data for 16 index traits included in the NTM index, which includes the YSS index. The selection of the DFS SNPs was undertaken within each breed according to *p*-values of a single-marker regression model while considering functional annotations and linkage disequilibrium between SNPs (Brøndum et al., 2015). Before the imputation, 54K genotypes were subjected to quality control using the minor allele frequency (MAF) threshold of 0.05. Bulls genotyped with 54K and the custom chips were imputed to 54K + DFS using FImpute software (Sargolzaei et al., 2014). Additionally, another set of WGS SNPs (147 SNPs) were selected from GWAS by Wu et al. (2017) for survival index (YSS_SNPs). The genotypes of these SNPs for the bulls in this study were imputed using the 1,000 bull genome data as reference and using the Minimac3 v.2.0.1 software (Das et al., 2016). The SNP-wise imputation accuracy was measured as the Pearson correlation between observed and imputed genotypes (coded as 0, 1, or 2) and the proportion of correctly imputed genotypes to all imputed genotypes (i.e., concordance). Only SNPs with both correlation and concordance higher than 0.80 were used in genomic prediction. Ultimately, 39,803 SNPs in the 54K chip, 1,541 DFS_SNPs, and 98 YSS_SNPs were kept for genomic prediction, with 22 SNPs overlapped between DFS and YSS_SNPs. The average imputation accuracy for SNPs used in genomic prediction was 0.977 for standard LD chip to 54K, 0.980 for DFS_SNPs, and 0.923 for YSS_SNPs, while concordance was 0.960 for standard LD chip to 54K, 0.962 for DFS_SNPs, and 0.955 for YSS_SNPs.

Of the 39,803 SNPs in the 54K chip used for the genomic prediction, 130 SNPs (LET_SNP) were within recessive lethal haplotypes reported by Wu et al. (2019) in the Nordic Holstein. The study of Wu et al. (2019) reported a total of 11 haplotypes of which nine were completely homozygous-deficient while two had significantly lower homozygotes observed than expected.

### Phenotypes

The traits included in the analyses were four different definitions of YSS (sub-traits) and an index trait (YSS index) derived from these four sub-traits. The sub-traits were as follows:

i) Bull period 1 (BP1): Bull calf survival day in the period 1–30 days;
ii) Bull period 2 (BP2): Bull calf survival day in the period 31–183 days;
iii) Heifer period 1 (HP1): Heifer calf survival day in the period 1–30 days;
iv) Heifer period 2 (HP2): Heifer calf survival day in the period 31–458 days.

Calf death and survival during each period were recorded as 0 and 1, respectively. Calves slaughtered, exported, or with missing records were recorded as missing. The YSS index was calculated by combining the estimated breeding values (EBVs) for the sub-traits, i.e., BP1, BP2, HP1, and HP2, by the Nordic Cattle Genetic Evaluation center (NAV, Denmark), which were weighted by their relative economic values and standardized in terms of mean and standard deviation (Pedersen et al., 2015).

De-regressed proof (DRP) derived from official EBV was used as the pseudo phenotype in the genomic prediction. The official EBVs were calculated using linear models by the Nordic Cattle Genetic Evaluation center as described in NAV (Nordic Cattle Genetic Evaluation) (2017). DRPs were derived using the official EBVs based on the standard method described in Jairath et al. (1998) and implemented using the mix99 program (Strandén, 2015).

**FIGURE 1:**
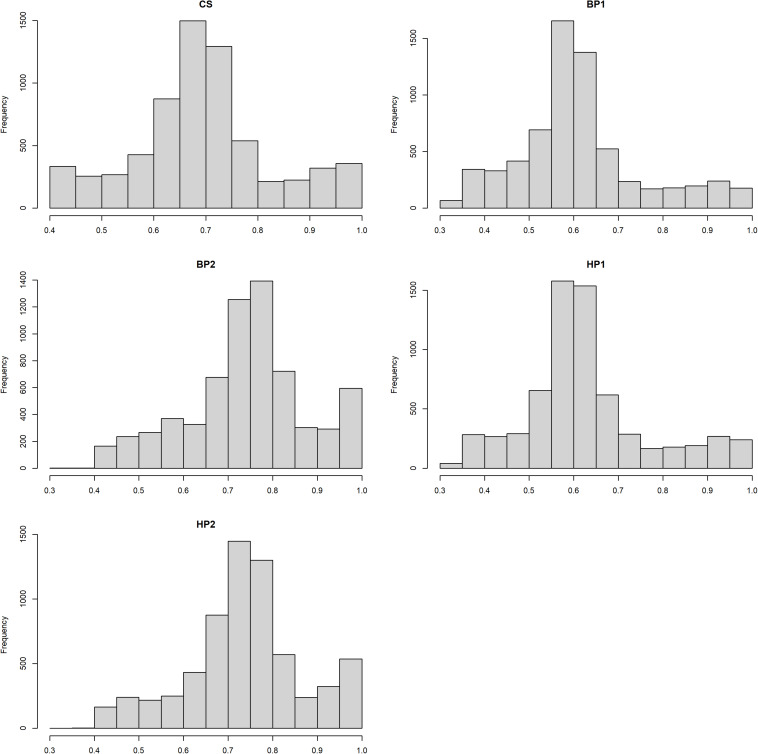
Histogram plots showing distributions of the de-regressed proof (DRP) reliabilities for the different traits.

The reliability of DRP was calculated as:

(1) $$r_{D\,R\,P\,i}^{2}=\frac{E\,D\,C_{i}}{E\,D\,C_{i}+\lambda^{\prime }}$$

where $\lambda =\frac{4-h^{2}}{h^{2}}$. The *EDC_i_* was the effective daughter contribution of *i*^*th*^ bull, and *h*^2^ was the heritability for each trait as used in the official Nordic Cattle Genetic Evaluation (Pedersen et al., 2015). The heritability estimates and mean DRP reliability for each trait are given in Table 1, and histogram plots showing reliability distributions are presented in Figure 1.

**TABLE 1:** Heritability estimates and mean reliability of DRPs used in the genomic prediction of the young stock survival traits.

Trait	*h*^2^*	Mean DRP reliability
YSS Index	0.014	0.698
BP1	0.007	0.611
BP2	0.027	0.742
HP1	0.009	0.626
HP2	0.011	0.737

*Heritability estimates used in the official Nordic evaluations (Pedersen et al., 2015). BP1, Bull period 1; BP2, Bull period 2; DRP, de-regressed proof; HP1, Heifer period 1; HP2, Heifer period 2; YSS, young stock survival.

### Statistical Analysis

Implemented prediction models included linear mixed model using pedigree-based best linear unbiased prediction (PBLUP)- or genomic best linear unbiased prediction (GBLUP)-based relationships. Different scenarios were investigated to study the effect of adding selected WGS SNPs and modeling recessive lethal SNPs on prediction reliability. These include:

(i) Only using 54K;
(ii) 54K plus YSS_SNPs (54K + YSS);
(iii) 54K plus DFS_SNPs (54K + DFS);
(iv) 54K plus YSS_SNPs and DFS_SNPs (54K + YSS + DFS);
(v) Reduced 54K (minus SNPs in recessive lethal haplotypes), plus YSS_SNPs and DFS_SNPS, and the model considered random regression on genotype code of LET_SNPs (54K^∗^ + YSS + DFS + LET).

In addition, two approaches of integrating the selected SNPs were assessed. Accordingly, one-component model pooling the selected WGS SNPs together with the 54K SNPs as one genetic component and two-component model considering 54K SNPs and selected WGS SNPs as two separate genetic components were implemented and compared for prediction accuracy.

The PBLUP model fitted was:

(2) y=1⁢μ+Za+e

where y is the vector of DRPs; **1** is the vector of ones; μ is the overall mean; **a** is the vector of additive genetic effects; **Z** is the incidence matrix relating **a** to phenotypes; and **e** is the vector of random residuals. It was assumed that $\text{a}∼\text{N}\,\left(0,\text{A}\,\sigma_{\text{a}}^{2}\right)$ and $\text{e}∼\text{N}\,\left(0,\text{D}\,\sigma_{\text{e}}^{2}\right)$. The **A** was the additive relationship matrix constructed from the pedigree that traced genotyped animals five generations back and included a total of 16,763 animals. The **D** is a diagonal matrix with elements$d_{i}=\frac{1-r_{D\,R\,P\,i}^{2}}{r_{D\,R\,P\,i}^{2}}$ for each bull *i* to account for heterogeneous residual variances due to differences in reliability of DRPs ($r_{D\,R\,P\,i}^{2}$) calculated as in Eq. 1.

The following one-component GBLUP models were fitted:

(3) y=1⁢μ+Zg+e

where **g** is the additive genetic effect with$g∼\text{N}\,\left(0,\text{G}\,\sigma_{\text{a}}^{2}\right)$, where **G** is the genomic relationship matrix (GRM) constructed using SNPs described in the different scenarios of adding WGS SNPs (YSS, DFS, or YSS + DFS) on the conventional 54K, while the remaining terms of the model are as described in model 2.

Additionally, a one-component GBLUP model considering random regression on the genotype code of the recessive lethal SNPs was implemented:

(4) $$y=1\,\mu +\text{Mb}+{\text{Zg}}^{*}+\text{e}$$

where **M** is a matrix of genotype code (0, 1, or 2) for recessive lethal SNPs with dimension of 6,558 (number of individuals) by 130 (number of recessive lethal SNPs), **b** is the vector of random regression coefficients on genotype code of recessive lethal SNPs (n = 130), and g^*^ is the random additive genetic effect based on GRM constructed using all SNPs (54K + YSS + DFS) excluding SNPs within recessive lethal haplotypes. The random regression coefficient b is assumed to be normally distributed:$\text{b}∼\text{N}\,\left(0,\text{I}\,\sigma_{\text{b}}^{2}\right)$, where **I** is an identity matrix and $\sigma_{\text{b}}^{2}$ is the variance of the regression coefficient estimates. In addition to the one-component models, genomic breeding values were also predicted using a two-component GBLUP model that accounted for the difference between effects of the 54K SNPs and effects of selected WGS SNPs. The two-component model for the 54K and WGS data was:

(5) $$y=1\,\mu +{\text{Zg}}_{54\,K}+{\text{Zg}}_{\mathrm{WGS}}+\text{e}$$

Additionally, a two-component model considering random regression on the genotype code of the recessive lethal SNPs was implemented:

(6) $$y=1\,\mu +\text{Mb}+{\text{Zg}}_{54\,K^{*}}+{\text{Zg}}_{\mathrm{WGS}}+\text{e}$$

where **M** and **b** are as described in model 4, **g**_54K^*^_ is the additive genetic effect based on GRM constructed with 54K SNPs excluding the SNPs within recessive lethal haplotypes, **g_WGS_** is the random genetic effect based on GRM constructed WGS SNPs (either DFS or YSS GWAS SNPs, or both, depending on the considered scenario).

An additional three-component GBLUP model was run to estimate the proportion of genomic variance explained by the SNP sets, i.e., 54K, YSS_SNPs, and DFS_SNPs by extending model 5 as follows:

(7) $$y=1\,\mu +{\text{Zg}}_{54\,\text{K}}+{\text{Zg}}_{\text{YSS}}+{\text{Zg}}_{\text{DFS}}+\text{e}$$

The proportion of the genomic variance explained by each SNP set of the three-component GBLUP model was then computed as:

(8) $$\%var_{S\,N\,P\,s\,e\,t_{i}}=\frac{\sigma_{S\,N\,P\,s\,e\,t_{i}}^{2}}{\sigma_{t\,o\,t\,a\,l}^{2}}\times 100,$$

where $\sigma_{S\,N\,P\,s\,e\,t_{i}}^{2}$ was the additive genetic variance estimated based on the GRM corresponding to each SNP set (54K, DFS, and YSS), and $\sigma_{t\,o\,t\,a\,l}^{2}$ was the total genomic variance computed as:

(9) $$\sigma_{t\,o\,t\,a\,l}^{2}=\sigma_{54\,K}^{2}+\sigma_{Y\,S\,S}^{2}+\sigma_{D\,F\,S}^{2}$$

All GRMs used for the different scenarios were calculated using the first method presented by VanRaden (2008), and SNP allele frequencies for building GRMs were calculated directly from the SNP data.

All models were implemented using the DMU software (Madsen and Jensen, 2013).

### Computation of Prediction Reliabilities

The studies of Wu et al. (2017, 2019) used part of the current dataset (bulls born on or before the year 2009) to detect the WGS markers for YSS and the recessive lethal haplotypes, respectively. Therefore, the validation set in the current study consisted of only bulls born after the year 2010 (*n* = 1,312), and the rest was used as the training population (*n* = 5,246).

Reliability of genomic prediction was computed as the squared correlation between estimated breeding values (GEBVs) and DRP divided by the average reliability of DRP for the bulls in the validation population. For the two-component GBLUP models, the total GEBV for each individual was computed by summing together the breeding values from the two components. Bias of prediction was measured as the regression coefficient of DRP on the estimated breeding values for the bulls in the validation population. Reliability and bias were then compared among different models.

For the model considering random regression on genotype codes of recessive lethal alleles, effects of the recessive lethal alleles from the random regression coefficients were added to the GEBVs to calculate the correlation with DRP and subsequently compute the reliability.

In addition, model fit for the different models was assessed and compared using the Akaike information criteria (AIC; Akaike, 1974).

## Results

### Proportion of the Genetic Variance Explained by the Different Single-Nucleotide Polymorphism Sets

**FIGURE 2:**
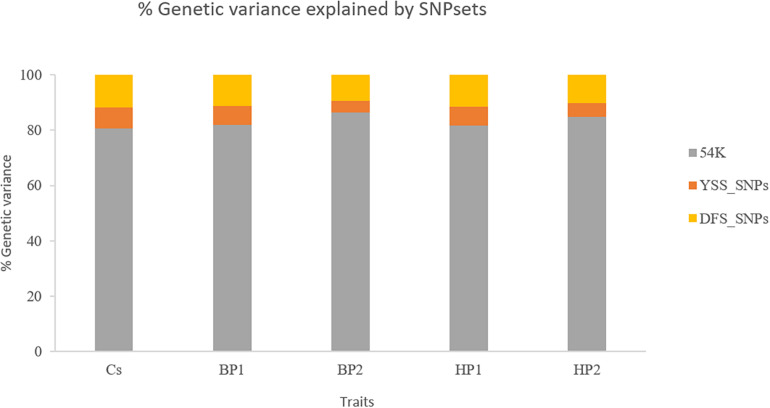
Percentages of the total genetic variance explained by the different single-nucleotide polymorphism (SNP) sets (54K, YSS_SNPs, and DFS_SNPs) in the different traits.

Figure 2 presents the percentages of total genomic variance explained by the different SNP sets, i.e., 54K SNPs, YSS_SNPs, and DFS_SNPs, in the different YSS sub-traits and the index trait. In general, at least 80% of the total genetic variance in all the traits is explained by the SNPs in the standard 54K chip. On average, the YSS_SNPs explained 6% of the genetic variation, while the DFS_SNPs explained 11%. Across the traits, the proportion of total genetic variance explained by YSS_SNPs (4.2%) and DFS_SNPs (9.5%) was lowest for BP2, which was 5% and 10.2% for YSS_SNPs and DFS_SNPs, respectively.

### Genomic Prediction Reliabilities and Bias

Table 2 presents genomic prediction accuracies using PBLUP and the GBLUP models that use different SNP sets. In general, across all scenarios, prediction reliability was lowest in the YSS index trait compared to the four sub-traits used to calculate the index trait. Among the sub-traits, prediction accuracies were higher for bull and heifer period 1 (BP1 and HP1) compared to the traits in period 2 (BP2 and HP2). For all the traits, the various GBLUP models resulted in higher prediction accuracies compared to the PBLUP model. An average gain in reliability of 16 percentage points was obtained using relationships derived from the 54K SNPs compared to using relationships derived from pedigree.

**TABLE 2:** Genomic prediction accuracies from PBLUP and GBLUP models.

Trait	PBLUP	GBLUP one-component					GBLUP two-component			
			
		54K	54K + YSS	54K + DFS	54K + YSS + DFS	54K* + YSS + DFS + LET	54K + YSS	54K + DFS	54K + YSS + DFS	54K* + YSS + DFS + LET
YSS Index	0.100	0.272	0.274	0.275	0.276	0.276	0.278	0.269	0.271	0.271
BP1	0.236	0.376	0.378	0.379	0.381	0.381	0.388	0.373	0.375	0.375
BP2	0.180	0.332	0.332	0.333	0.334	0.334	0.333	0.330	0.331	0.332
HP1	0.267	0.404	0.406	0.404	0.404	0.404	0.413	0.391	0.393	0.393
HP2	0.140	0.308	0.308	0.307	0.308	0.308	0.309	0.302	0.303	0.303

*54K + YSS = Conventional 54K SNPs plus SNPs from GWAS on YSS (YSS_SNPs).*

*54K + DFS = Conventional 54K SNPs plus SNPs from GWAS on all traits in Nordic total merit index (DFS_SNPs).*

*54K + YSS + DFS = Conventional 54K SNPs plus YSS_SNPs and DFS_SNPs.*

54K* + YSS + DFS + LET = Reduced 54K (minus SNPs in recessive lethal haplotypes), plus YSS_SNPs (YSS) and DFS_SNPs and the model considered random regression on genotype code of SNPs in recessive lethal haplotypes (LET_SNPs).

*BP1, Bull period 1; BP2, Bull period 2; GBLUP, genomic best linear unbiased prediction; GWAS, genome-wide association study; HP1, Heifer period 1; HP2, Heifer period 2; PBLUP, pedigree-based best linear unbiased prediction; SNP, single-nucleotide polymorphism; YSS, young stock survival.*

Comparison among the GBLUP models using different SNP sets in one- or two-component models indicates no or only marginal improvements in prediction accuracies compared to using only the 54K data. On average over the five traits, the improvement in prediction reliability obtained from adding the YSS_SNPs in the one-component model compared to prediction using only the 54K markers was 0.12 percentage points. Similar results were obtained when the 54K marker set was augmented with DFS_SNPs in the one-component model. Fitting both the YSS_SNP sets and DFS_SNPs together with the 54K markers in the one-component model resulted in an average gain in reliability of 0.22 percentage points compared to the prediction using only 54K markers. Additional consideration of random regression on genotype code of recessive lethal alleles in this model did not result in further improvement of prediction reliability. Among the two-component GBLUP models, addition of the YSS_SNPs resulted in an average improvement of 0.58 percentage points compared to the prediction with only 54K SNPs. Addition of the rest of SNP sets (DFS, DFS + YSS) using the two-component GBLUP resulted in slightly lower prediction reliability compared to the model using only 54K.

Table 3 presents the bias in predicting the breeding values across the different models. Regression coefficients were generally close to 1.00 across the different models. Between the different traits, regression coefficient for BP1 and HP1 were generally lower compared to BP2 and HP2 as well as the YSS index trait. For these traits (BP1 and HP1), the one-component GBLUP resulted in slightly less bias compared to the two-component GBLUP model. In addition, model fit for the different scenarios assessed with the AIC is presented in Table 4. Generally, the GBLUP models had lower AIC values compared to the PBLUP models across all the traits. Hence, the GBLUP models tend to have better fit to the data compared to the PBLUP models, which is in agreement with the overall performance of the two models in prediction accuracy. Among the different GBLUP models, the AIC values computed for the different scenarios were quite comparable.

**TABLE 3:** Regression coefficients^a^ of DRP on prediction.

Trait	PBLUP	GBLUP one-component					GBLUP two-component			
			
		54K	54K + YSS	54K + DFS	54K + YSS + DFS	54K* + YSS + DFS + LET	54K + YSS	54K + DFS	54K + YSS + DFS	54K* + YSS + DFS + LET
YSS Index	0.976	1.027	1.026	1.027	1.026	1.022	1.003	1.005	1.000	0.998
BP1	0.976	0.892	0.893	0.891	0.891	0.888	0.891	0.866	0.865	0.863
BP2	1.046	0.953	0.954	0.954	0.955	0.952	0.955	0.954	0.954	0.952
HP1	0.968	0.886	0.887	0.885	0.885	0.883	0.884	0.864	0.863	0.862
HP2	1.045	0.968	0.969	0.967	0.967	0.964	0.965	0.963	0.963	0.960

*^*a*^Standard errors of regression coefficients across the scenarios = (0.059–0.092).*

*54K + YSS = Conventional 54K SNPs plus SNPs from GWAS on young stock survival (YSS_SNPs).*

*54K + DFS = Conventional 54K SNPs plus SNPs from GWAS on all traits in Nordic total merit index (DFS_SNPs).*

*54K + YSS + DFS = Conventional 54K SNPs plus YSS_SNPs and DFS_SNPs.*

54K* + YSS + DFS + LET = Reduced 54K (minus SNPs in recessive lethal haplotypes), plus YSS_SNPs (YSS) and DFS_SNPs and the model considered random regression on genotype code of SNPs in recessive lethal haplotypes (LET_SNPs).

*BP1, Bull period 1; BP2, Bull period 2; DRP, de-regressed proof; GBLUP, genomic best linear unbiased prediction; GWAS, genome-wide association study; HP1, Heifer period 1; HP2, Heifer period 2; PBLUP, pedigree-based best linear unbiased prediction; SNP, single-nucleotide polymorphism; YSS, young stock survival.*

**TABLE 4:** Akaike information criteria (AIC) for the different models implemented.^a^

Trait	PBLUP	GBLUP one-component					GBLUP two-component			
			
		54K	54K + YSS	54K + DFS	54K + YSS + DFS	54K* + YSS + DFS + LET	54K + YSS	54K + DFS	54K + YSS + DFS	54K* + YSS + DFS + LET
YSS Index	−26.99	−45.18	−45.17	−45.16	−45.16	−45.13	−45.12	−45.14	−45.12	−45.10
BP1	−27.65	−39.86	−39.85	−39.84	−39.84	−39.82	−39.81	−39.82	−39.81	−39.79
BP2	−22.16	−44.98	−44.97	−44.96	−44.96	−44.93	−44.94	−44.95	−44.94	−44.91
HP1	−26.26	−40.75	−40.74	−40.73	−40.72	−40.70	−40.70	−40.70	−40.69	−40.67
HP2	−24.00	−44.96	−44.95	−44.94	−44.94	−44.91	−44.92	−44.93	−44.92	−44.89

*^*a*^x10^3^.*

*54K + YSS = Conventional 54K SNPs plus SNPs from GWAS on young stock survival (YSS_SNPs).*

*54K + DFS = Conventional 54K SNPs plus SNPs from GWAS on all traits in Nordic total merit index (DFS_SNPs).*

*54K + YSS + DFS = Conventional 54K SNPs plus YSS_SNPs and DFS_SNPs.*

54K* + YSS + DFS + LET = Reduced 54K (minus SNPs in recessive lethal haplotypes), plus YSS_SNPs (YSS) and DFS_SNPs and the model considered random regression on genotype code of SNPs in recessive lethal haplotypes (LET_SNPs).

*BP1, Bull period 1; BP2, Bull period 2; GBLUP, genomic best linear unbiased prediction; GWAS, genome-wide association study; HP1, Heifer period 1; HP2, Heifer period 2; PBLUP, pedigree-based best linear unbiased prediction; SNP, single-nucleotide polymorphism; YSS, young stock survival.*

## Discussion

### Genomic Prediction Accuracies for Young Stock Survival Traits

In general, prediction accuracies for the YSS index trait and the sub-traits were low in our study across scenarios. Our findings are however, in line with reported prediction accuracies in the literature for calf and YSS traits defined in various periods. In a previous study, genomic prediction accuracies ranging between 0.15 and 0.30 were reported for maternal calf survival in different parities for the Canadian Holstein (Abo-Ismail et al., 2017).

Accurate genomic prediction of survival traits in cattle is difficult (van der Heide et al., 2020), as the traits are affected by a combination of environmental factors such as farm management as well as non-additive genetic effects such as recessive lethal gene effects (Gebreyesus et al., 2020).

Across the studied YSS traits, relatively higher prediction accuracies were observed for BP1 and HP1 compared to the YSS index trait and the other two sub-traits. Although the heritability estimates (Table 1) for all the traits studied here are among the lowest of the dairy cattle traits (Pedersen et al., 2015), heritability for BP1 and HP1 was even lower compared to the other sub-traits and the index trait. Similarly, DRP reliabilities were slightly lower for BP1 and HP1. Therefore, the slightly higher prediction reliability for BP1 and HP1 was contrary to our expectations. DRP reliability is the function of number of records used to estimate the EBVs and heritability of the traits. Across the studied traits, heritability is quite low and differences in heritability between the traits are small. Therefore, the slight differences in average DRP reliabilities between the studied traits might be due to differences in numbers of observations used to predict the EBVs of the bulls for different traits in the official Nordic cattle evaluations.

### Benefits of Incorporation of Selected Variants on Genomic Prediction Reliability

In our study, integration of additional selected WGS SNPs and recessive lethal haplotypes resulted in negligible improvement in genomic prediction reliability for YSS index and the four sub-traits. Previous studies reported some gains in genomic prediction accuracies from additional variants selected from WGS data using GWAS, functional annotation, and pathway analysis, depending on the trait and population studied [e.g., Brøndum et al. (2015), van den Berg et al. (2016), Liu et al. (2019)]. Gains in genomic prediction reliability from integration of additional selected WGS SNPs partly depend on the genetic architecture of the traits and consequently the proportion of variation explained by the selected SNPs (Hayes et al., 2010). In the literature, while additional WGS SNPs improved genomic prediction accuracies for some traits, often marginal improvement is reported for others. Liu et al. (2019) for instance reported increases in prediction accuracies for milk production traits in the Danish Jersey from addition of selected WGS SNPs but lack of improvement in prediction reliability for fertility and only marginal improvement for mastitis. Brøndum et al. (2015) reported increases in prediction reliability of up to 5 percentage points for milk production traits in Nordic Holstein and Red populations, while improvement of reliability was negligible for fertility. Similar results were reported in the study of Veerkamp et al. (2016) where genomic prediction with the addition of a selected set of WGS variants for protein yield (PY), somatic cell score (SCS), and interval from first to last insemination led to negligible improvement in prediction reliability. In the current study, neither of the SNP sets, i.e., DFS_SNPs and YSS_SNPs, led to improvement in prediction reliability of the YSS traits. The DFS_SNPs explained on average 11% of the genomic variance for the studied traits compared to an average of 6% explained by the YSS_SNPs. However, the higher proportion of genomic variance explained by the DFS SNPs in contrast to the YSS SNPs could be merely due to the difference in the number of SNPs in the two sets. The DFS SNPs were selected based on relevance to multiple traits including production, disease, and calving traits. Moreover, the NTM index, which is based on several traits that include the YSS trait, was considered in the selection of the DFS SNPs (Brøndum et al., 2015). However, the main emphasis, in terms of weights, was placed on milk production traits compared to fitness traits such as fertility, mastitis, and other disease traits, as well as the NTM index. On the other hand, the YSS_SNPs reported by Wu et al. (2017) were selected based on GWAS for YSS index specifically; therefore, improvements in prediction reliability were to be expected compared to the DFS SNPs. However, the YSS_SNPs included only 98 SNPs that might make it difficult to explain a sizable proportion of the genetic variation for polygenic traits such as YSS (Wu et al., 2017).

Additionally, the effects of selected variants might be somehow underestimated in this study due to the use DRPs as response variable rather than raw phenotypes for the survival traits. This might specially be of relevant impact to the models that include the effect of recessive lethal alleles rather than those incorporating the selected WGS SNPs, as these were selected based on GWASs using DRPs as response variable (Brøndum et al., 2015; Wu et al., 2017).

### One-Component vs. Two-Component Genomic Best Linear Unbiased Prediction Models

It has also been shown that the effect of integrating selected variants on the reliability of genomic prediction might depend on whether or not the effects of these variants have been weighted appropriately in the models (Raymond et al., 2018). In the traditional GBLUP model, the contribution of genetic markers to the genomic relationship is the same. In this context, Sørensen et al. (2014) suggested an extension of the GBLUP model to allow differentiation among the markers through a genomic feature BLUP (GFBLUP) approach. In GFBLUP, variants are categorized according to biological information, such as chromosomes, genes, or biological pathways, so that the random genetic effect in the GBLUP model can have more than one component. Implementation of such an approach to integrate selected variants has shown improvement in genomic prediction reliability compared to integrating them using the traditional one-component GBLUP approach. Gebreyesus et al. (2019) reported substantial increases in genomic prediction reliability in different Holstein cattle populations for milk fatty acid composition traits by incorporating selected variants through the three-component GBLUP model compared to pooling all variants in one GRM. Similar improvements using the two-component GBLUP model were reported in pigs (Sarup et al., 2016; Song et al., 2019).

Contrary to these previous findings, there was no difference in prediction reliability from integrating the selected WGS SNP sets through the two-component model compared to the one-component GBLUP in our study. Multiple-component GBLUP model involves simultaneous estimation of more parameters in addition to those estimated in a one-component model. Thus, gains from multiple-component GBLUP, *vis-à-vis* one-component, can only be expected if addition of information from the additional component(s) is substantial enough to offset the extra uncertainty due to more parameters to be estimated in the multiple-component analysis.

## Conclusion

In this study, we hypothesize that incorporation of WGS variants selected based on GWAS and variants within recessive lethal haplotypes might improve the reliability of genomic prediction for YSS traits. We tested our hypothesis using one- or two-component GBLUP models. Contrary to our hypothesis, the results showed negligible improvements by incorporation of such variants in genomic prediction accuracies for the YSS index trait and the four sub-traits. The results highlight the difficulty in genetic evaluation for polygenic traits with very low heritability such as the YSS traits and the need for further studies to explore additional information including the genomic information beyond SNP variants to improve the prediction reliability for these traits.

## Data Availability Statement

The data analyzed in this study are subject to the following licenses/restrictions: Phenotypic and genomic data used in this study are property of the industry partners that contributed to the study. Requests to access these datasets should be directed to the corresponding author.

## Ethics Statement

Ethical review and approval was not required for the animal study because all procedures to collect the DNA samples followed the protocols approved by the National Guidelines for Animal Experimentation and the Danish Animal Experimental Ethics Committee, and hence, no specific permission was required. Written informed consent was obtained from the owners for the participation of their animals in this study.

## Author Contributions

GG processed the data, implemented the analyses, and drafted the manuscript. GSu conceived the study and contributed to the discussion of the results. ML contributed to the interpretation and discussion of the results. GSa acquired funding and contributed to the discussion of the results. All authors contributed to the article and approved the submitted version.

## Conflict of Interest

The authors declare that the research was conducted in the absence of any commercial or financial relationships that could be construed as a potential conflict of interest. The handling editor declared a past co-authorship with one of the authors ML.

## Footnotes

1 www.nordicebv.info
